# Colorectal anastomotic healing: why the biological processes that lead to anastomotic leakage should be revealed prior to conducting intervention studies

**DOI:** 10.1186/s12876-015-0410-3

**Published:** 2015-12-21

**Authors:** Joanna W. A. M. Bosmans, Audrey C. H. M. Jongen, Nicole D. Bouvy, Joep P. M. Derikx

**Affiliations:** Department of Surgery, Maastricht University Medical Centre, Maastricht, The Netherlands; NUTRIM School for Nutrition, Toxicology and Metabolism, Maastricht University, Maastricht, The Netherlands; Pediatric Surgical Center Amsterdam, Emma Children’s Hospital AMC/VUMC, P.O.Box 22660, 1100 DD Amsterdam, The Netherlands

**Keywords:** Animal research, Anastomotic healing, Anastomotic leakage, Colorectal anastomoses, Colorectal surgery, Healing process, Wound healing

## Abstract

**Background:**

Anastomotic leakage (AL) remains the most dreaded complication after colorectal surgery and causes high morbidity and mortality. The pathophysiology of AL remains unclear, despite numerous studies that have been conducted on animals and humans, probably due to the undetermined healing process of colorectal anastomoses. Increasing basic knowledge on this healing process may shed more light on causal factors of AL, and additionally reduce the quantity and accelerate the quality of experimental studies. In this debate article, our aim was to provide different perspectives on what is known about the colorectal healing process in relation to wound healing and AL.

**Discussion:**

Since knowledge on anastomotic healing is lacking, it remains difficult to conclude which factors are essential in preventing AL. This is essential information in the framework of humane animal research, where the focus should lie on Replacement, Reduction and Refinement (3Rs). While many researchers compare anastomotic healing with wound healing in the skin, there are substantial recognized differences, e.g. other collagen subtypes and different components involved.

**Summary:**

Based on our findings in literature as well as discussions with experts, we advocate stop considering anastomotic healing in the gastrointestinal tract and cutaneous healing as a similar process. Furthermore, intervention studies should at least address the anastomotic healing process in terms of histology and certain surrogate markers. Finally, the anastomotic healing process ought to be further elucidated – with modern techniques to achieve 3Rs in animal research - to provide starting points for potential interventions that can prevent AL.

## Background

Anastomotic leakage (AL) remains the most dreaded complication after colorectal surgery and is associated with high morbidity and mortality [1–3]. Despite extensive research into possible risk factors and numerous studies aiming to minimize and/or prevent consequences of AL, it remains unclear why some colorectal anastomoses leak while others do not. As a consequence, surgeons have difficulties predicting surgical outcome [4, 5].

AL is considered the result of disrupted anastomotic healing [6]. Obviously, in the aetiology of AL, the technical aspect should not be neglected, however, even a perfect anastomosis made by the most skilled surgeon can still develop dehiscence. The pathophysiology causing this complication remains unknown despite devoted effort [6–8], most likely due to the fact that the anastomotic healing process is not yet fully understood. Nevertheless, this lacuna in knowledge has not retained researchers from investigating prophylactic measures that may prevent AL to occur, using all kinds of animal models and interventions to achieve their goal.

A recent systematic review showed that animal research on AL is of poor quality and still increasing, contrary to societal aims. [9] The principle of the 3Rs was already developed over 50 years ago, but it is questionable if this actually has led to Replacement, Reduction and Refinement (3Rs) of animals in scientific research. Is it ethical to continue performing prophylactic intervention studies on animals when the physiology of anastomotic healing is still not fully determined?

A search including “*the effect of … on anastomotic healing*” in PubMed, retrieves 471 articles (September 2015). However, the majority of these articles lack in providing details on anastomotic healing. The conclusions are mainly based on a reduction in AL rate or an increase in bursting pressure. Although generally accepted in the literature, we wonder if these outcome measures truly are surrogate markers for anastomotic healing. Furthermore, many researchers draw direct parallels between anastomotic healing and cutaneous wound healing, but are these processes indeed comparable or should they be considered as two separate entities?

Additionally, it is difficult to come up with new treatment strategies or preventive measures in order to decrease AL when the pathophysiology of anastomotic healing is not known. For example, if it was not for Barry Marshall infecting himself with H. Pylori, we still would not have known that peptic ulcers can be cured with the use of antibiotics [10]. The focus of research on bowel anastomoses should therefore be redirected from prophylactic interventions tested on animals towards studies that unravel the processes of normal anastomotic healing. This should include the identification of essential factors and possible deficiencies in these factors that lead to a disruptive healing process and consequently AL. One of the key elements in identifying disruptive factors is the detection of risk factors. Risk factors for the development of AL are for example older age, male gender, malnutrition and operative time as several studies have demonstrated [11–13]. However, to date, no predictive score of patients’ risk of AL have led to a decision-making tool for choice of protective measures in the clinical setting [14, 15].

In this debate article, we aim to address the discussion that is still on going in the research field of anastomotic healing by providing views from different perspectives. Without crucial information on the pathophysiology of anastomotic leakage, we believe that this is a complication that will remain to exist in the clinical setting. That is why we want to advocate performing more basic studies on elucidating the physiology of gastrointestinal anastomotic healing prior to conducting intervention studies, which is actually where the majority of both clinical and experimental studies focuses on at the moment.

## Discussion

To date, no consensus has been reached among researchers regarding

1. which layer is most important in anastomotic healing;
2. if gastrointestinal healing can be compared to cutaneous healing;
3. if bacteria play a role in the pathogenesis of anastomotic leakage; and
4. if surrogate markers truly provide information regarding anastomotic healing,

mainly due to a lack of knowledge on these subjects.

1. Which layers of the colon are of importance in anastomotic healing?

The bowel wall of the colon consists of four layers: mucosa, submucosa, muscularis propria and serosa. Whenever a colorectal resection is performed, all these layers are transsected and an anastomosis can be constructed. Although surgeons in the past opted for suturing either the serosa (Lembert 1826), the serosa together with the mucosal surface (Czerny 1881) or the submucosa (Halstead 1887), modern techniques for creating an anastomosis such as stapler devices make no distinction and involve all these layers [16]. No evidence was found to demonstrate any superiority of stapled over hand sewn techniques in colorectal anastomosis surgery, regardless of the level of anastomosis [17]. Although it is stated that the submucosa of the bowel is a tough fibrous layer consisting mainly of collagen and elastin fibers and has the greatest tensile strength of the four layers [18], the configuration of the suture bite is an infrequent study subject. Both full-thickness and sero-submucosal sutures seem to be sufficient to anatomically apposition both sides of the bowel, thereby promoting wound healing [19]. It can be hypothesized that the submucosa is of great importance in anastomotic healing. Indeed, this layer is the source of fibroblasts that become active after gastrointestinal surgery and start to deposit collagen. Daams et al. showed that the healing of everting anastomoses in an experimental model occurred by formation of a fibrotic cap at the serosal side, that formed a matrix for fibroblasts [20]. This is in accordance with the first classic stage of wound repair: inflammation. Here, a fibrin matrix is formed as part of haemostasis which serves as a scaffold for infiltrating cells [21]. However, the role of the mucosa is completely neglected when the submucosa is considered the most important layer of the bowel wall regarding healing. In the early 90s, it was demonstrated that an anastomosis causes a deep and long lasting reduction in energy metabolism, especially in the mucosa and the muscle layers [22]. Recent evidence suggests a role for bacteria in the pathogenesis of AL, which occurs when the healing process is disrupted [23, 24]. Since bacteria house in the mucus of the colon, the function of the mucosa should not be disregarded and may even play a more important role than is yet recognized. In addition, we have demonstrated that Muc2 knockout mice are more prone to the development of AL than control mice, indicating that a normal mucus layer facilitates the anastomotic healing process [25].

Furthermore, macrophages in the gastrointestinal mucosa represent the largest pool of tissue macrophages in the body and a long-lasting macrophage absence or dysfunction impairs anastomotic healing [26, 27]. Macrophages are one of the main factors in the inflammatory response, and based on their behaviour, this response is either pro-inflammatory (M1) impairing wound healing or anti-inflammatory (M2) promoting wound healing; a shift in the M2/M1 index can influence the outcome of anastomotic healing [28]. The interaction between intraluminal content and the several layers of the bowel wall with their separate cell types and function may be key in unravelling the healing process.

To sum up, all layers seem to play a role in anastomotic healing. The submucosa consists of connective tissue and has the greatest tensile strength of the four layers. Moreover, the serosa seems to be important in providing a matrix for fibroblasts, while the interaction between bacteria, mucus and the mucosal layer also seem important to maintain homeostasis in which anastomotic healing can occur. The focus of research into anastomotic healing should lie on transmural evaluation of the healing process and interaction between layers of the bowel wall.

2) Gastrointestinal healing versus skin healing

Already in 1997, it was stated by Thornton that unlike cutaneous healing, healing of the gastrointestinal tract - more specifically the intestinal anastomosis - is anatomically obscured from inspection, allowing the surgeon to judge the success of the operation only on the patient’s parameters of general wellbeing [6]. Not only is this bothersome in daily practice, it may also be part of the explanation that knowledge of gastrointestinal healing is lagging behind compared to wound healing of the skin. The classic phases of wound healing (inflammation, proliferation and remodelling) have been studied extensively in skin [21] and many researchers describe gastrointestinal healing in terms of these phases [29, 30]. It is true that these 3 phases exist in all types of tissue, however, there are significant differences between skin and gastrointestinal healing [18]. These differences relate to collagen and collagenase activity, wound strength and wound environment. First of all, collagen subtypes in the gastrointestinal tract (I, III, V) are produced by fibroblasts and smooth muscle cells compared to solely I and III produced by fibroblasts in the skin. Collagenase activity plays an important role in the healing of anastomoses, where a high activity causes collagen lysis that results in low anastomotic strength early after the formation of an anastomosis [31]. Wound healing is far more rapid in the gastrointestinal tract than in the skin despite the potential risks, such as shear stress, bacteria that may affect anastomotic healing and changes in vascular perfusion that are more abundant in the intestinal environment [6, 18]. As previously stated, the serosal layer plays an important role in terms of strength of the wound while there is no equivalent component in cutaneous healing. Not only are the components not similar, the reaction of both tissues is also not the same. For example, Törkvist and colleagues tried to block CD18-dependent neutrophil infiltration to improve wound healing and concluded that neutrophils may influence the wound healing process differently in specific organs, based on diverse results in skin and intestinal tract [32]. One of the explanations for their different results may lie in differences between cutaneous and intestinal collagen synthesis [33], however, also the skin flora and gut microbiota vary completely, which can play an important role in differences in wound healing [34, 35].

In conclusion, gastrointestinal – and more specifically anastomotic – healing differs significantly from cutaneous healing. Although there are similarities, especially in the phases of wound healing, one cannot directly compare these two processes and therefore, gastrointestinal anastomotic healing should be considered a separate entity that needs to be investigated in more detail.

3) The involvement of bacteria in the pathogenesis of anastomotic leakage

From experimental studies, evidence suggests a positive effect of antibiotics on the strength of colonic anastomosis [36, 37]. This implicates that when certain bacteria are being inhibited, this actually enhances anastomotic healing. Alverdy and colleagues have been investigating this hypothesis for some time and proposed a significant role for bacteria in the pathophysiology of AL in 2013 [4]. In the past years, they have shown that virulent bacteria with high collagenase activity may contribute to the development of AL [23]. The interaction between intraluminal content and the layers of the bowel wall may be key in the physiology of anastomotic healing. Since bacteria reside in the colonic mucus layer, they could be the reason why mice without normal mucus composition suffered more from AL, as stated previously. In addition, it has been shown that butyrate, a short-chain fatty acid (SCFA) produced by microbiota can strengthen colonic anastomoses in rats ( [38]. Already in 1973, it was demonstrated by Levison that SCFAs inhibited in vitro growth of P. Aeruginosa [39] exactly the pathogen that was later identified as being able to transform into tissue destroying phenotype with high collagenase activity [24]. It seems undeniable that bacteria play a role in the pathogenesis of AL. However, clinical implications for these findings are lacking. It remains to be elucidated whether eliminating bacteria by perioperative antibiotics or promoting the growth of certain species with probiotics can improve anastomotic healing. Eliminating faeces from the colon prior to surgery can be achieved with mechanical bowel preparation. Nevertheless, mechanical bowel preparation – that was traditionally used together with oral antibiotics – did not show any beneficial effect on the occurrence of AL according to several randomized trials and was therefore abandoned. However, just recently, an attempt has been made to clarify the effect of mechanical bowel preparation with or without antibiotics [40], using a large retrospective cohort. Since the use of oral antibiotics alone has not been investigated in the majority of these studies, there is not enough evidence to conclude anything regarding oral antibiotics independently of mechanical bowel preparation.

In summary, given the available data, there seems to be a role for bacteria in the pathogenesis of anastomotic leakage. More research is needed to completely elucidate this role and the interaction of microbiota with specific cells and their excreted products at the anastomotic site.

4) Surrogate markers of anastomotic healing

The most frequently used surrogate marker of anastomotic healing in animal models is bursting pressure (BP). Although there are many methods to test BP, it all comes down to inflating or filling the bowel segment with the anastomotic site and measuring the intraluminal pressure at which either air or fluid leakage is observed at the site of the anastomosis. While this outcome measure has been debated for possibly disrupting tissue samples, making histological evaluation difficult and though critics have stated that BP is not a relevant indicator since it can not be applied for anastomoses that already have a leak [41], it is still considered appropriate by researchers in the field since it offers an actual surrogate outcome: anastomotic strength (Delphi consensus conducted June 2015, data submitted). Quantitative comparison of BP is not valid between studies, since the protocols and instruments vary tremendously; therefore, only BP values can be directly compared between experimental groups in the same study [7]. Other mechanical tests such as measurement breaking strength or tensile strength are extensively criticized for either being not sensitive enough to measure in the early healing phase and because of its technical difficulties [42]. Histological healing parameters are often reported in experimental studies regarding gastrointestinal anastomoses, mostly by grading scales regarding factors based on cutaneous wound healing such as inflammatory cell infiltration, fibroblast activity, collagen deposition and vascularity or neoangiogenesis [30, 43–45]. These parameters are considered helpful in evaluating the general wound healing process at the anastomotic site. However, a limitation may be that to investigate the true leak in the anastomotic line, histological evaluation has to be performed right at the spot of the leak.

In wound healing, fibroblasts replace the provisional matrix that is established during inflammation with collagen-rich granulation tissue making collagen an interesting marker for anastomotic healing [18]. Therefore, other potential surrogate markers are analyses that measure collagen content, synthesis and degradation [46]. Quantification of collagen is often performed by measuring hydroxyproline content, since this amino acid is found in few proteins other than collagen [7]. Although hydroxyproline content is considered informative about the amount of collagen, it does not provide information on collagen subtypes, the maturity of the collagen and therefore not necessarily the tissue strength, since this lies more in the type and quality of collagen present in the anastomotic tissue [47–49]. Sirius Red staining combined with digital imaging to analyse the percentage of collagen type I and III can demonstrate the ratio between young and mature collagen [39, 50]. A disadvantage of this technique - and all immunohistochemistry analyses - is that is a non-quantitative method, however, it does locate the specific site in the tissue where the deposition of collagen occurs and current computer imaging techniques can aid in the quantification of different subtypes of collagen [51]. Collagen degradation can be mediated through Matrix Metalloproteinases (MMP) resulting in loosening of the matrix that may result in AL [52]. This collagenase activity of MMPs – especially MMP9 is associated with AL – can be measured by quantitative gelatin zymography [23, 39, 53, 54]. Again the statement of the spatial and regional context of measuring bacteria and inflammatory mediators applies here; in grinding up tissues you are getting the average of the entire tissue sample, while the most important measure is likely to be right at the site of the necrosis and leak. However, it has been demonstrated that it is possible to distinguish between changes in the composition of the intestinal microbiota associated with anastomotic tissue and microbiota associated with luminal contents [55].

In summary, frequently used surrogate markers for anastomotic healing in animal models are bursting pressure, tensile strength and a generic histological examination. Other additional analyses are used to answer specific research question, but new techniques are necessary to obtain more insight in the anastomotic healing process.

## Summary

An on going increase in studies regarding anastomotic healing can be found in literature. Despite extensive observational and experimental research in animal models and in humans, the incidence of AL has remained unchanged. We believe that this is largely because causal factors leading to colorectal AL are yet still not recognized.

The current hypothesis is that AL has a complex multifactorial pathophysiology, where at least ischemia, bacteria and inflammation are involved [23]. Ideally, a technique that can provide an overview of the healing process at different time points, differentiate between inflammatory markers and various cell types and zoom in at the site of the leakage in the anastomotic line can help to unravel the biological process of anastomotic healing. Furthermore, intestinal wound healing is continuously compared with cutaneous wound healing in literature, despite evident differences. Besides, many studies focus on the effect of a certain intervention on the AL rate, without studying the biochemical process of anastomotic healing first. An international summit on intestinal AL concluded that research into the pathogenesis of AL could be advanced markedly by performing additional analyses in human anastomotic tissues during and after surgery [56]. The use of human tissue (or microbiota) will lead to a reduced demand of experimental research, thereby reducing the numbers of animals that are being used nowadays. Furthermore, with current technologies, we are capable of replacing animals with, for example, organoids to investigate the molecular process of intestinal healing in more detail or to use less animals since high resolution mass-spectrometry imaging techniques or digital holographic microscopy can provide more information for the assessment of gastrointestinal wound healing [57–59].

## Conclusions

We have argued that the ill-defined pathogenesis of AL is a direct consequence from the largely unexplained, complicated biological process of anastomotic healing. Anastomotic healing should be completely elucidated – similar to cutaneous wound healing – in order to develop interventions that may stimulate anastomotic healing and subsequently prevent AL. This can positively influence the quality and decrease the quantity of animal research on AL in the short term and hopefully reduce the clinical burden of AL in the long term.

## Abbreviations

3Rs: Replacement, Reduction and Refinement
AL: anastomotic leakage
BP: bursting pressure
MMP: matrix metalloproteinase
